# The Involvement of Voltage-Operated Calcium Channels in Somato-Dendritic Oxytocin Release

**DOI:** 10.1371/journal.pone.0025366

**Published:** 2011-10-20

**Authors:** Vicky A. Tobin, Alison J. Douglas, Gareth Leng, Mike Ludwig

**Affiliations:** Centre for Integrative Physiology, University of Edinburgh, Edinburgh, United Kingdom; Centre National de la Recherche Scientifique, University of Bordeaux, France

## Abstract

Magnocellular neurons of the supraoptic nucleus (SON) secrete oxytocin and vasopressin from axon terminals in the neurohypophysis, but they also release large amounts of peptide from their somata and dendrites, and this can be regulated both by activity-dependent Ca^2+^ influx and by mobilization of intracellular Ca^2+^. This somato-dendritic release can also be *primed* by agents that mobilise intracellular Ca^2+^, meaning that the extent to which it is activity-dependent, is physiologically labile. We investigated the role of different Ca^2+^ channels in somato-dendritic release; blocking N-type channels reduced depolarisation-induced oxytocin release from SONs *in vitro* from adult and post-natal day 8 (PND-8) rats, blocking L-type only had effect in PND-8 rats, while blocking other channel types had no significant effect. When oxytocin release was primed by prior exposure to thapsigargin, both N- and L-type channel blockers reduced release, while P/Q and R-type blockers were ineffective. Using confocal microscopy, we found immunoreactivity for Ca_v_1.2 and 1.3 channel subunits (which both form L-type channels), 2.1 (P/Q type), 2.2 (N-type) and 2.3 (R-type) in the somata and dendrites of both oxytocin and vasopressin neurons, and the intensity of the immunofluorescence signal for different subunits differed between PND-8, adult and lactating rats. Using patch-clamp electrophysiology, the N-type Ca^2+^ current density increased after thapsigargin treatment, but did not alter the voltage sensitivity of the channel. These results suggest that the expression, location or availability of N-type Ca^2+^ channels is altered when required for high rates of somato-dendritic peptide release.

## Introduction

Many neurons release a wide variety of signalling molecules from their soma and dendrites to autoregulate their activity, communicate with adjacent neurons and glia, and modulate afferent nerve ending activity [1]–[4].

In magnocellular neurons of the supraoptic (SON) and paraventricular nuclei, oxytocin and vasopressin are released from somata and dendrites, with important consequences [5], [6]. In particular, somato-dendritic oxytocin release plays an essential role in the milk-ejection reflex, co-ordinating the intermittent synchronous activation of oxytocin neurons that underlies pulsatile oxytocin secretion [7], [8]. Somato-dendritic oxytocin and vasopressin release is also prominent during early postnatal development, when it is important for the modelling of dendritic morphology [9].

Like the release of conventional neurotransmitters, oxytocin and vasopressin secretion from axon terminals in the neurohypophysis depends on depolarisation-evoked Ca^2+^ entry through high-voltage-activated Ca^2+^ channels. N-, P/Q- and (in vasopressin neurons only) R-type channels regulate action-potential dependent secretion from the terminals, while L- channels are present at the terminals but play little role in this [10]. Similarly, at most synapses in the CNS neurotransmitter release is governed by N and/or P/Q type channels [11], [12]. Somato-dendritic release of oxytocin and vasopressin also depends on the entry of extracellular Ca^2+^ [13], [14] and L-, P/Q-, N- and R-type channels are expressed on the somata and dendrites of magnocellular neurones [15]–[19].

Dendritic release of oxytocin and vasopressin can also be evoked by agents that mobilise intracellular Ca^2+^ stores, and this can occur independently of electrical activity, and hence independently of secretion from the neurohypophysis [20], [21]. Dendritic release can also be evoked by electrical activity, but for this to occur *in vivo*, the neurons must first be “primed”. Priming involves a long-lasting increase in the releasability of vesicles in the soma and dendrites (no priming occur in the axon terminals) [20], [22], and is thought to involve translocation of vesicles to release sites at the plasma membrane [23] adjacent to voltage-gated Ca^2+^ channels.

Dendritic oxytocin and vasopressin release can be primed *in vitro* by exposing the isolated SON to mobilisation of intracellular Ca^2+^ by thapsigargin or cyclopiazonic acid, and is seen as a late emerging and prolonged potentiation of depolarisation evoked peptide release [20], [22]. We hypothesised that this might be achieved in part by a change in Ca^2+^ entry via voltage operated Ca^2+^ channels.

High-voltage activated Ca^2+^ channels have an α_1_-subunit, which forms the Ca^2+^-selective membrane pore, and auxiliary subunits which modulate channel properties such as inactivation and channel targeting; Ca_V_1.2 and 1.3 form L-type channels and Ca_V_2.1, 2.2 and 2.3 form P/Q-, N- and R-type channels respectively [24].

Here we used selective Ca^2+^ channel blockers to identify their contributions to depolarisation-induced oxytocin release and to whole-cell Ca^2+^ currents. We have also used fluorescence immunocytochemistry to describe the α_1_-subunits distribution in oxytocin and vasopressin neurons and to determine changes in subunit expression at times of high dendritic release (lactation and postnatal day 8 (PND-8)) and after thapsigargin-induced priming.

## Results

### Involvement of different Ca^2+^ channels in somato-dendritic peptide release

**Figure 1:**
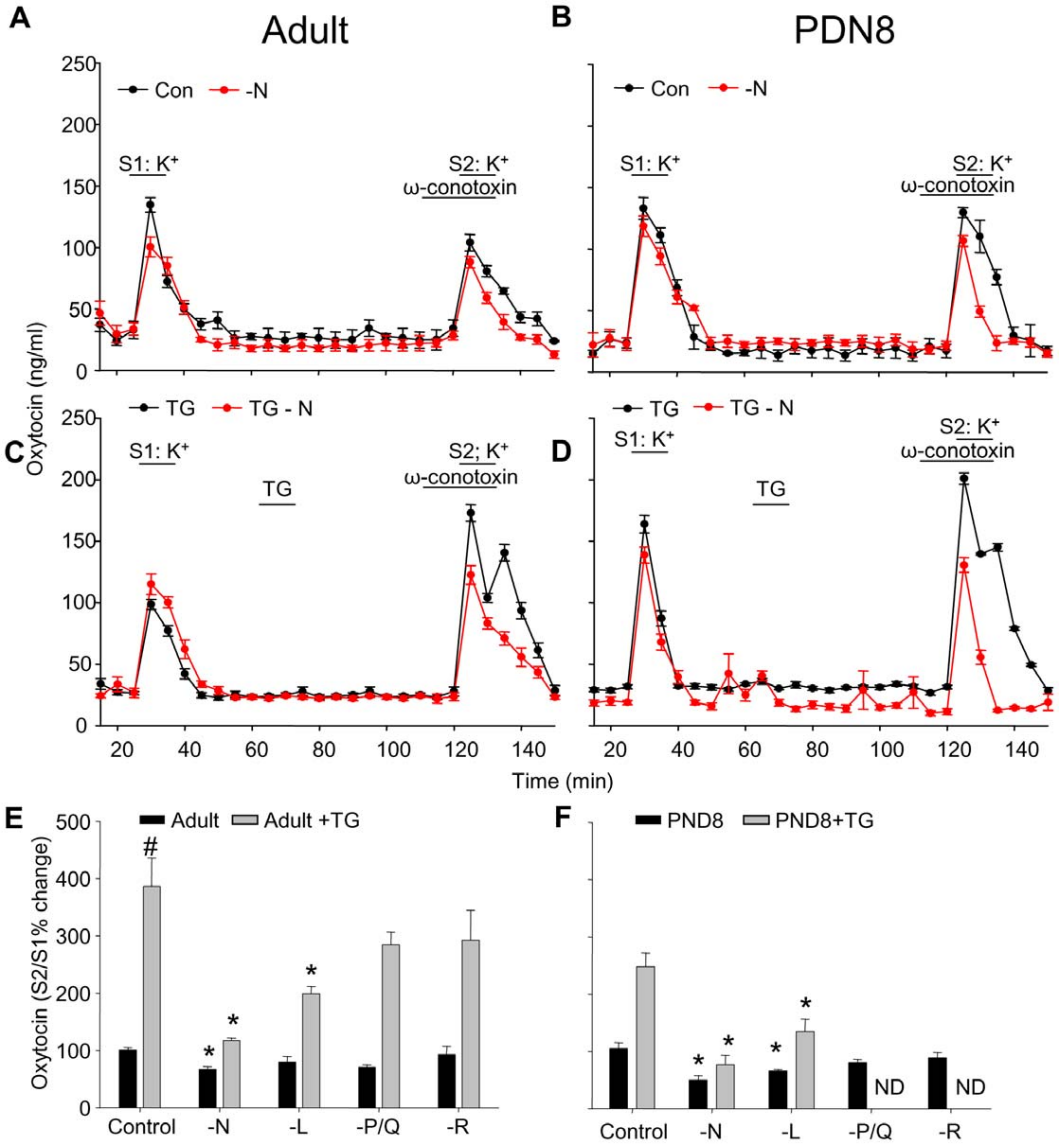
Effects of VOCC toxins on oxytocin release from SONs *in vitro.* Blocking N-type VOCC in SONs from adult (**A**) PDN-8 (B), thapsigargin- (TG)-treated adult rats (**C**) and TG-treated PDN-8 rats (D) shows a decrease in amplitude and duration of the potassium stimulated response. Stimulated release was enhanced after pretreatment with 0.2 µM TG as previously described (**C D** black lines) (Ludwig et al., 2002); this was prevented by blocking N-type Ca^2+^ channels (red lines). Effects of blocking different VOCC on the S2:S1 oxytocin response in SONs from adult and adult TG-treated rats (E) and PND-8 rats and TG-treated PDN-8 rats (**F**). Means±S.E.M., *n* = 6 per group, # *P*<0.05 vs first S1, * *P*<0.05 vs control, one-way ANOVA followed by Dunn's *post-hoc* test.

Explants of the SON responded to two repeated depolarisations (S1 and S2: 50 mM K^+^) with similar amounts of oxytocin release (Fig. 1A,B; controls are shown in black lines). The Ca^2+^ channel toxins were applied for 20 min, 10 min before and 10 min during the S2 stimulation. The effects of Ca^2+^ channel toxins were quantified as the release in response to S2 (in the presence of toxin) expressed as a percentage of the response to S1. Each toxin was tested in a separate set of experiments. The data for toxin and vehicle experiments were analysed together (using an ANOVA followed by Dunn's *post-hoc* test) for experiments in adult rats, adult rats treated with thapsigargin, and PND-8 rats.

The N-type channel blocker ω-conotoxin GV1A reduced stimulus-evoked oxytocin release from the SON of both adult and PND-8 rats (Fig. 1A,B; S2:S1, 50±8% and 67±5% respectively, *P*<0.05), while treatment with the vehicle control (0.1% DMSO) had no significant effect (adult and PND-8 S2:S1 101±4% and 105±11; Fig. 1A,E and Fig. 1B,F). The L-type blocker nicardipine also reduced release from the SON in PND-8 rats (S2:S1, 66±3%, *P*<0.05) but not significantly in adult rats (S2:S1, 80±10%, N.S.). P/Q- and R-type blockers had no significant effect on release in any group (Fig. 1B–D).

In SON neurons, thapsigargin induces an increase in intracellular [Ca^2+^] lasting ∼10 min [25] accompanied by an immediate but small increase in somato-dendritic oxytocin and vasopressin release; in addition, thapsigargin provokes a persistent change in the releasability of peptides from the somata and dendrites (*priming*). This is apparent as significantly increased peptide release in response to a later depolarising stimulus; it emerges at least 15 min after thapsigargin treatment and lasts for at least 90 min [20]. We tested whether this priming might, in part, reflect a change in Ca^2+^ entry via voltage-activated Ca^2+^ channels.

Confirming previous studies [20], pre-exposure of adult rat SONs to thapsigargin for 10 min produced a marked enhancement of oxytocin release evoked by a subsequent depolarisation (applied 30 min after thapsigargin; Fig. 1C, black line; Fig. 1E, S2:S1, 386±50%; *P*<0.05, *t* test vs vehicle controls). Thapsigargin treatment also primed oxytocin release in SONs from PND-8 rats (Fig. 1D black line; Fig. 1F S2:S1 248±23%; *P*<0.05, t-test vs vehicle controls). This primed response was reduced in the presence of the N-type blocker in SONs from both adult and PND-8 rats (S2:S1: 117±5% and 77±17% respectively; Fig. 1C,D red lines; Fig. 1E,F; *P*<0.05 ANOVA). The primed response was also reduced (though to a lesser extent) by the L-type blocker nicardipine (adult and PND-8 S2:S1: 199%±13% and 135±22, *P*<0.05, ANOVA, Fig. 1E,F). P/Q- and R-type blockers had no significant effect in SONs of adult rats with or without thapsigargin pretreatment (Fig. 1E). There were also no significant effects of blocking P/Q and R-type Ca^2+^ channels in PND-8 SONs (their effects were not determined (ND) after TG treatment, Fig. 1F). As highlighted in Figures 1A–D, the effect of N-type channel blockade on depolarisation-induced oxytocin release in both PND-8 and adult SONs with and without priming is a small decrease in the amplitude of the response but a large decrease in its duration.

Thus depolarisation-induced oxytocin release from adult rat SONs depends on Ca^2+^ influx particularly via N-type channels, and thapsigargin-induced priming may involve a change in the activity and/or numbers of these channels.

### Immunohistochemical characterisation of VOCC α_1_ subunits in SON sections

**Figure 2:**
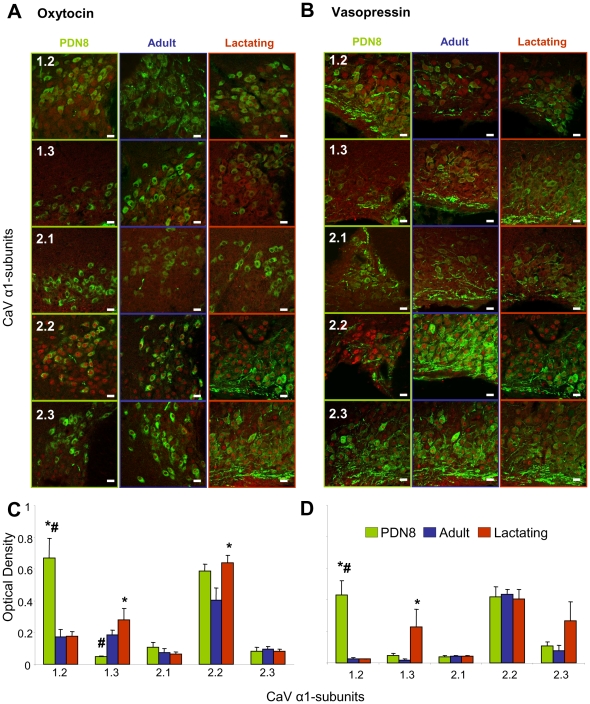
Ca^2+^ channel distribution in tissue sections of SON. (**A**) Immunostaining for Ca_v_ subunits (red staining) and oxytocin (**A**) or vasopressin (**B**) (green staining) in SON sections from PND-8, adult, and lactating rats. Average optical density for the subunits normalised by the optical density of (**C**) oxytocin and (**D**) vasopressin (n = 5). No labelling was detected when primary antibodies were omitted or incubated with a five-fold excess of control immunogen before being exposed to the tissues (not shown). Means±S.E.M. vs adult, # *P*<0.05 vs lactating rats, one-way ANOVA followed by Holm-Sidak *post-hoc* test. Scale bars, 20 µm.

To examine changes in the expression of Ca_v_ subunits at times of high secretory demand, we compared Ca_V_ α-subunit immunoreactivity in SON sections from PND-8 and lactating rats with sections from adult rats. Specificity of Ca_v_ α-subunit immunoreactivity was assessed by pre-absorption controls. Each antibody was pre-incubated with five-fold concentration of immunogen used to raise the antibody (provided by Alomone) before being applied to representative sections and this resulted in no fluorescent signal (data not shown). In adult rats (Fig. 2A,B), immunoreactivity for all five α_1_-subunits was present in the somata and dendrites of both vasopressin and oxytocin neurons. To compare signals between groups, the optical density of Ca_v_ subunits were measured in oxytocin or vasopressin immunoreactive regions of the SON. The optical density of subunits in these regions showed age- and physiological state-dependent differences (Fig. 2C,D). In areas of the SON defined by immunoreactivity for either oxytocin or vasopressin, the signal for Ca_v_1.2 was stronger in PND-8 rats than in adult and lactating rats (*P*<0.05, ANOVA). On the other hand, the Ca_v_1.3 signal was weaker in oxytocin (but not vasopressin) areas of PND-8 rats (*P*<0.05 vs adults, ANOVA), and stronger in both oxytocin and vasopressin regions from lactating rats (*P*<0.05 vs adults, ANOVA). The Ca_v_2.2 signal was not changed in vasopressin regions, but in oxytocin areas it was stronger in PND-8 and during lactation (*P*<0.05 vs adults, ANOVA).

Thus, during lactation, when central and peripheral oxytocin release is increased, there is also an increased expression of Ca_v_1.3 and 2.2, which define L and N-type channels respectively in oxytocin-immunoreactive regions. In PND-8 rats, where there is high dendritic secretion of both oxytocin and vasopressin, there is higher expression of Ca_v_1.2 (which also defines L-type channels) in both oxytocin- and vasopressin-rich regions of the SON, and higher expression of Ca_v_2.2 in oxytocin-rich regions. These changes include signal throughout the neuron somata and dendrites both in the cytoplasm and at the plasma membrane.

### Immunohistochemical characterisation of VOCC α_1_ subunits in single SON neurons

**Figure 3:**
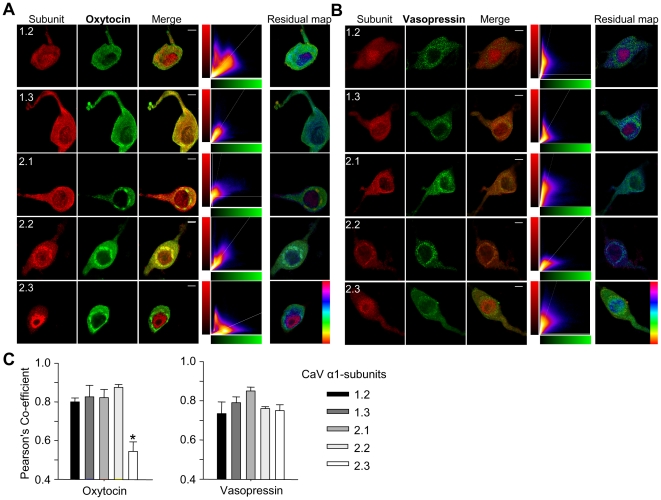
Ca^2+^ channel distribution in isolated magnocellular neurons. Immunostaining for Ca_v_ α1 subunits (red) in acutely-dispersed oxytocin (**A**) and vasopressin neurons (**B**) (green). Immunostaining for all subunits was observed in the soma and dendrites, in both in the cytoplasm and at the plasma membrane. Immunostaining for oxytocin and Ca_v_2.1 and 2.2 also showed strong perinuclear staining. The merged images show areas of coincidence in yellow (third column). Scatter plots (fourth column) show co-variance of subunit signal with the oxytocin signal for each voxel The residual map corresponds to weighted residuals from the line fit to the scatter plot, thus indicating fluorescent channel covariance (hue from –1 to 1, with cyan corresponding to zero residual). (**C**) Quantification of covariance data of oxytocin or vasopressin and one of the five α1-subunits using Pearson's coefficient values (n = 5 per group). Means±S.E.M. * *P*<0.05, one-way ANOVA followed by Holm-Sidak *post-hoc* test vs Ca_v_2.2. Scale bars 10 µm.

In other neuroendocrine cells there is evidence for co-localisation of Ca^2+^ channel subtypes with secretory vesicles at the plasma membrane [26]. We used fluorescence immunohistochemistry and confocal microscopy to investigate associations of Ca^2+^ channels and peptide-containing vesicles in acutely dissociated SON neurons (Fig. 3). Again, the specificity of the Ca_v_ α-subunit antibodies was assessed by pre-incubating each antibody with a five-fold (w/v) of the immunogen used to raise the antibody (provided by antibody supplier–Alomone). In all cases, pre-incubation of the antibody with the immunogen resulted in no fluorescent signal (data not shown).

In oxytocin neurons, although a punctate oxytocin signal was present in all cytoplasmic regions, the density was consistently highest in the perinuclear region (Fig. 3A). In vasopressin neurons, punctate immunoreactivity for vasopressin was present at a relatively constant average density throughout the cytoplasm in the soma and proximal dendrites (Fig. 3B).

Punctate immunoreactivity for all α_1_-subunits was present in both oxytocin and vasopressin neurons in their somata and proximal dendrites (Fig. 3A,B). In both cell types, the Ca_v_1.3 and 2.1 subunits are evenly distributed throughout the cytoplasm, whereas the signal for Ca_v_2.2 was strongest in the perinuclear region. Ca_v_1.2 and 2.3 subunits were present throughout the cytoplasm, but there was also a strong nuclear signal in both oxytocin and vasopressin neurons.

We quantitatively analysed the colocalisation of each channel subunit with oxytocin, vasopressin, and a Golgi marker. The degree of colocalisation can be inferred by correlating the intensity of the subunit signal with the oxytocin or vasopressin signal for every voxel (scatterplots in Fig. 3A,B). The best fit line was then used to calculate the Pearson's coefficient of variation, which ranges from 0.5 (random co-localisation) to 1.0 (perfect co-localisation). The residual maps show the spatial distribution of the Pearson's coefficient of variation (i.e. where colocalisation is strongest, represented in cyan and weakest, in red). For colocalisation with oxytocin, the mean Pearson' coefficient of variation was highest for Ca_v_2.2 (0.88±0.02, n = 5, Fig. 3C), and lowest for Ca_v_2.3; the association of Ca_v_2.3 with oxytocin (0.54±0.05) was close to that expected for random colocalisation. The residual map for Ca_v_2.3 and oxytocin shows that the nucleus contains a relatively strong Ca_v_2.3 signal and a weak or absent oxytocin signal, while the subplasmalemal zone shows the converse. For Ca_v_2.2, the residual maps show some signal in the nucleus, but in all other areas of the cell there was relatively strong colocalisation with oxytocin. For vasopressin there was no significant difference in the Pearson' coefficient of variation for any of the subunits (Fig. 3C).

**Figure 4:**
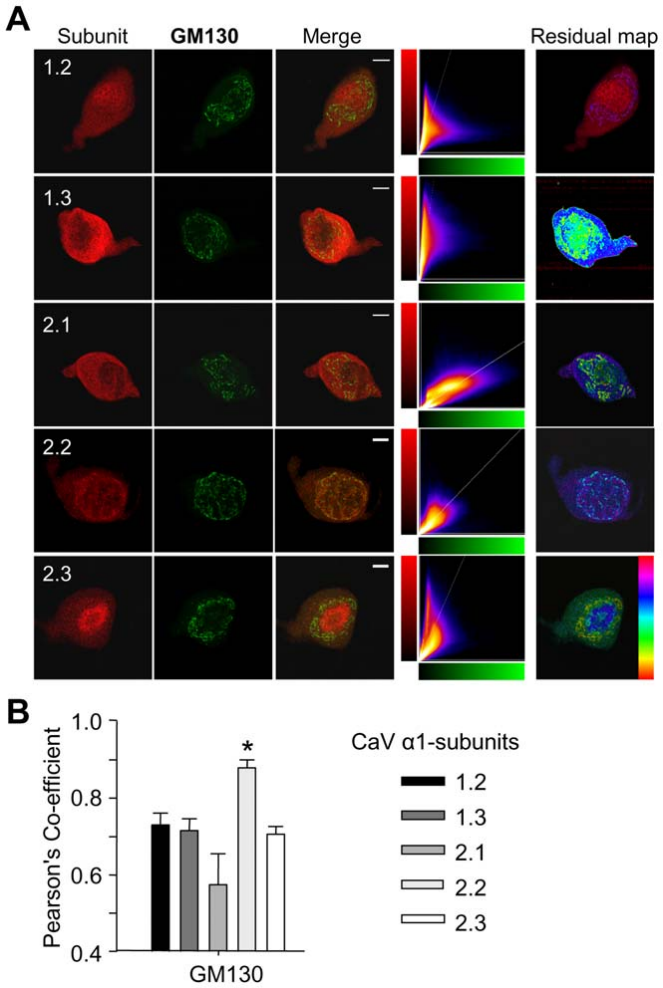
Ca^2+^ channel distribution in relation to the Golgi apparatus. (**A**) Immunostaining for Ca_v_ α1 subunits (red) and the Golgi marker GM130 (green) in acutely-dispersed supraoptic neurons. There was strong co-localisation of Ca_v_2.2 with GM130, a marker for the Golgi apparatus. No fluorescent labelling was detected when primary antibodies were omitted or incubated with a five-fold excess of control immunogen before being exposed to the cells (not shown). (**C**) Quantification of covariance data of GM130 and one of the five α1-subunits using Pearson's coefficient values (n = 5 per group) showed a significant difference between the co-localisation of GM130 and Ca_v_2.2 versus the other α1-subunits. Means±S.E.M. * *P*<0.05, one-way ANOVA followed by Holm-Sidak *post-hoc* test vs Ca_v_2.2. Scale bars 10 µm.

As Ca_v_2.1 and 2.2 subunits consistently appeared to have a strong perinuclear signal (Fig. 4A), we examined the association of subunits with the Golgi marker (cis-Golgi matrix protein; GM130). The Pearson's co-efficient for Ca_v_2.2 with GM130 was significantly greater than that between the Golgi and all other subunits (Fig. 4B, *P*<0.05).

Thus the subcellular distribution of Ca_v_2.2, which defines N-type channels, is similar to that of oxytocin immunoreactivity, with a particularly strong signal at the Golgi apparatus where oxytocin is packaged into dense-cored vesicles. In vasopressin neurons there is a much weaker colocalisation of Ca_v_2.2 with vasopressin, as vasopressin immunoreactivity is not as strongly expressed at the Golgi as oxytocin immunoreactive signal. The results suggest that even if the N-type channels are not expressed in the same vesicles as either peptide, that there may be a common pathway or control of the intracellular distribution and translocation of the N-type channels. Dense-cored vesicles in oxytocin and vasopressin magnocellular neurons have previously been demonstrated to be re-distributed towards the plasma membrane following a priming stimulus [23].

### Effect of priming on Ca^2+^ current densities

Changes in immunohistochemically identified α-subunits do not necessarily represent changes in the related Ca^2+^ currents. Therefore, we investigated changes in Ca^2+^ currents in isolated magnocellular neurons from adult rats using specific Ca^2+^ channel blockers before and after priming with thapsigargin.

**Figure 5:**
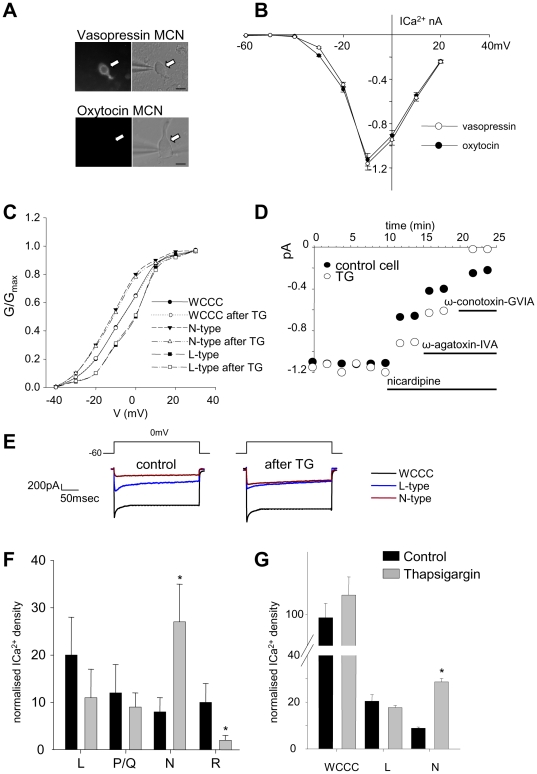
Effects of channel toxins on **Ca^2+^** currents in isolated SON neurons. (**A**) Isolated magnocellular neurons were identified as either vasopressin or oxytocin by their presence or absence of the GFP signal. Scale bar = 10 µm. (**B**) The current-voltage relationships show no difference between whole-cell calcium currents (WCCC) of vasopressin and oxytocin neurons (n = 20 each). (**C**) Normalized conductance (G/G_max_, fitted using Boltzmann equation) showed no significant change with or without TG. (**D**) Examples of two neurons (both oxytocin neurons), one vehicle treated (•) and one 50 min after pre-treating with thapsigargin (○). The steady-state Ca^2+^ current is plotted against time following treatment with channel toxins (in order: 1 µM nicardipine; 200 nM ω-agatoxin IV; 500 nM ω-conotoxin GVIA). The current measured after each toxin was subtracted from that evoked before each treatment to determine the current carried by L-, P/Q- and N-type channels. The remaining current was attributed to R-type channels. (**E**) Examples of N-, L- and WCCC elicited by voltage steps in controls and TG treated cells. (**F**) The resulting current density for each current type normalised for the peak maximal current was averaged (n = 5) to give the average current density from neurons treated with vehicle or thapsigargin. (**G**) In a subsequent experiment L- or N-type channel toxins were administered, alternating the order (n = 5 for each condition). Mean±S.E.M are shown and compared by t-test. *P<0.05 vs control.

Before being patched, neurons were treated with either vehicle (DMSO 0.1%) or thapsigargin (0.2 µM) for 30 min and identified as vasopressin or oxytocin cells by the presence or absence of an eGFP signal, respectively (Fig. 5A). Whole-cell calcium currents (WCCC) were evoked by voltage steps from −60 to +30 mV (in 10 mV increments) over 250 ms. The results showed that the current-voltage relationship was not different between oxytocin and vasopressin cells and thus data were pooled for analysis (Fig. 5B). These currents were normalised against the peak maximum current evoked by a depolarisation to −10 mV. These were used to express the mean normalised conductance (G/G_max_) at each potential. The data points were fitted using the following Boltzmann equation (see Methods, Fig. 5C).

V_0.5_ (which represents the half-maximal activation) and *k* (which represents the conductance slope) for WCCC, N-, and L-type Ca^2+^ currents were each compared with and without TG treatment and showed no significant difference (Fig. 5C). Thus TG treatment does not affect the activation kinetics for WCCC, N-, and L-type Ca^2+^ currents.

In addition, voltage-dependent currents were evoked by a voltage step from -60 mV to 0 mV for 250 ms, repeated three times every 5 min (Fig. 5E). The current amplitude in the last 10 ms of the step (steady-state current) was measured from the average current evoked from the three voltage steps. Steady-state current was measured before and after sequential applications of the channel blockers (1 µM nicardipine, 0.2 µM ω-agatoxin IVA, 0.5 µM ω-conotoxin GVIA and 0.02 µM SNX 482 or vehicle (0.1% DMSO). Once started, each Ca^2+^ channel blocker was continuously applied until the end of recording to ensure no effect of toxin wash-out. The amplitude of current carried by each channel was calculated as the difference between the amplitude of the current after the specific Ca^2+^ channel toxin treatment and the current before treatment (Fig. 5D). In five cells currents measured using the voltage-step protocol repeated three times every 5 min for 45 min without application of toxins showed no change in amplitude, indicating no run down in Ca^2+^ currents (dada not shown).

Acutely dissociated magnocellular neurons retain different lengths of dendrites and thus have different whole-cell Ca^2+^ currents, so we normalised Ca^2+^ currents by the capacitance measured for that cell, giving current densities (pA/pF). Initially we did not determine whether cells were oxytocin or vasopressin neurons, but both show thapsigargin-induced priming [20], [22]. The current densities of each channel type were averaged (Fig. 5F) and the effect of thapsigargin pretreatment on the inferred amplitude of current carried by each channel type was compared by unpaired *t*-test. In five control cells, the N-type channel blocker ω-conotoxin GVIA had the least effect on voltage-gated Ca^2+^ entry, while the L-type channel blocker nicardipine had the greatest effect (Fig. 5D, black circles). In five cells pre-treated with thapsigargin (0.2 µM for 10 min; 30–50 min before measurement of current densities), ω-conotoxin GVIA had a greater effect than in control cells (Fig. 5F, grey bars, *P*<0.05 vs control), with no significant differences in the effects of P/Q and L-type channel blockers. There was no significant difference in the average WCCC amplitude between control cells and thapsigargin-treated cells.

In an additional experiment, using identified vasopressin and oxytocin cells, again we found no significant difference in the WCCC. The L- and N-type channel toxins were applied in alternating order to exclude an effect of treatment order. As there was no effect of treatment order and no difference between cell types, the results were pooled. The results confirmed that, after TG treatment there was a significant increase in N-type Ca^2+^ current density, but no significant change in WCCC and the L-type current density (Fig. 5G).

Overall these results show that TG treatment results in an increase in Ca^2+^ current carried by the N-type Ca^2+^ channel. The lack of effect on channel activation kinetics suggests that TG treatment increases the number of N-type channels.

## Discussion

In the present study, we found that *in vitro* depolarisation-induced oxytocin secretion from adult SONs depends on Ca^2+^ influx particularly *via* N-type channels. As reported previously (and replicated in the present experiments), mobilisation of intracellular Ca^2+^ by thapsigargin results in a prolonged enhancement (‘priming’) of depolarisation-induced peptide release, and this is particularly marked for oxytocin [20]. We have previously shown that priming involves a reorganisation of the F-actin cytoskeletal network and translocation of peptide-containing vesicles to close to the plasma membrane [21], [27]. Priming is potentially an important general mechanism of neuronal plasticity; in the oxytocin system, priming has been proposed to underlie the functional reorganisation of the oxytocin system in lactating rats in response to suckling, giving rise to the ability of this system to generate synchronised bursts of electrical activity leading to the pulsatile oxytocin secretion that is essential for efficient milk let-down [8], [20]. Here, we found that thapsigargin-induced priming was accompanied by a significant increase in both the Ca^2+^ current carried by N-type channels and the extent to which depolarisation-evoked dendritic release depends on these channels.

We saw some contribution of L-type channels to evoked release in both control and thapsigargin-primed conditions in adult and PDN-8 rats, but no evidence of a significant contribution from P/Q- or R-type channels, although at the concentrations used all of the blockers had at least as great an effect as the N-type channel blocker ω-conotoxin GVIA on voltage-gated Ca^2+^ current. However, it should be noted that in these experiments we used high K^+^ as a depolarising stimulus, and in the neurohypophysis the contribution of L-type channels to secretion evoked by high K^+^ is greater than to secretion evoked by action potentials.

Previous studies have shown expression of L-, P/Q-, N- and R-type channels in both the somata-dendrites [10], [19] and nerve terminals of magnocellular neurones [15], [17], [28]–[30]. Thus, all of the Ca^2+^ channels that are expressed at the axon terminals are also expressed at the cell bodies, but with different biophysical properties; in particular, the N channels in the terminals inactivate rapidly [28], whereas those in the soma are slowly or non-inactivating [17].

In synapses in the CNS, the sites of neurotransmitter release are defined by active zones containing clusters of Ca^2+^ channels [31]. Localised zones of Ca^2+^ entry have been identified in dendritic spines of hippocampal CA3 neurons [32], [33], but there is no functional or morphological evidence for active zones in the soma, dendrites or axon terminals of vasopressin and oxytocin cells [34]. Indeed, in magnocellular neurons, exocytosis can apparently occur at *any* site on the cell surface–including even from undilated axons; from electron-microscopy studies it appears that the proportion of release observed from any of the compartments of magnocellular neurons (soma, dendrite, undilated axons, axonal swellings and nerve endings) simply reflects the number of vesicles in that compartment that are close to the cell membrane. If this is the case, how is it that exocytosis (from nerve terminals or dendrites) is so dependent on N-type channels, when this subtype is responsible for *less* voltage-gated Ca^2+^ entry than other subtypes? The likely answer is that, in magnocellular neurons as in other CNS neurons, N-type channels are strongly clustered at sites on the cell membrane, that at these sites there is a large but highly localised Ca^2+^ entry, and as a result these clusters define sites of exocytosis, but in magnocellular neurons these clusters are not localised to synapses, and occur in all parts of the neuron.

Different Ca_V_ subtypes show different subcellular distributions in neurons; Ca_V_2.1 and Ca_V_2.2 are mainly localised in axons and at synapses, while Ca_V_1 is mainly present in the soma and dendrites. Cytoskeletal elements are involved in the localisation of Ca_V_ to the plasma membrane [35]–[38]. In chromaffin cells, Ca_V_2 channels are inserted into the plasma membrane as part of the process of transforming growth cones into mature neurosecretory endings [39], [40]. In neuroblastoma cells, Ca_V_2.2 channels are held in a large reservoir of secretory granules at the Golgi and can be recruited to the plasma membrane during regulated secretion [41]. Recruitment of Ca^2+^ channels from internal stores to the plasma membrane has also been reported in bag cell neurons of *Aplysia californica* [42]–[44]. In hippocampal neurons, during long term potentiation, different Ca^2+^ channels (in particular Ca_V_2.2) are recruited to the plasma membrane to enhance neurotransmitter release, at specific synapses [45].

The sub-cellular distribution of Ca_v_2.2 was very similar to that of oxytocin, with a particularly strong signal at the Golgi apparatus where oxytocin is packaged into dense-cored vesicles. We also found an increased expression of Ca_v_2.2 in lactation, when there is a high demand for dendritic oxytocin release, and in PND-8 rats, where there is high dendritic secretion of both oxytocin and vasopressin. Ca_v_2.2, which defines N-type channels, might be co-packaged in peptide-containing vesicles, as suggested by one electron-microscopy study of the neurohypophysis [46]. Alternatively, separate vesicles containing Ca_v_2.2 may be tethered or transported by the same cytoskeletal and molecular motors as used by peptide-containing vesicles, as suggested by another electron microscopy study [47].

The results presented in this paper do not allow us to definitively state that N-type calcium channels are similarly re-distributed to the plasma membrane after thapsigargin treatment. However, we found the calcium current carried by N-type channels to be increased after thapsigargin. This could be due to an increase in numbers of channels at the membrane (by either increased synthesis or translocation of existing channels to the plasma membrane). The time between thapsigargin treatment and the measurement of increased calcium current carried by the N-type calcium channels argues against de novo synthesis of N-type calcium channels and we found the Ca_V_ 2.2 subunit to be strongly co-localised with a Golgi marker, unlike other Ca_V_ α1 subunits, suggesting a reserve pool of Ca_V_2.2 subunit. Or the increase could be due to an increased activity of existing N-type channels meaning greater calcium conductance per channel. We have examined this possibility by conducting additional experiments, comparing the kinetics of the calcium currents carried by N-type calcium channels (the activation-voltage relationships), suggesting that thapsigargin treatment resulted in an increase in N-type channels at the plasma membrane. Thus, we hypothesis that a reserve pool of N-type Ca^2+^ channels may be recruited to the plasma membrane in response to the priming signal to increase activity-dependent peptide release.

In conclusion, in magnocellular neurons, N-type channels appear to be more functionally associated with somato-dendritic secretion than other Ca^2+^channels, as blocking N-type channels was most effective in inhibiting high K^+^-induced oxytocin secretion both before and after priming, and priming exclusively increased Ca^2+^ influx via N-type channels. Changes in N-type channel availability at the plasma membrane may be crucial in translating a change in action potential firing to somato-dendritic peptide release.

## Materials and Methods

### Animals

Female Sprague-Dawley and Sprague-Wistar rats homozygous for an enhanced green fluorescent protein–vasopressin fusion gene (eGFP-vasopressin) [48] rats were housed under controlled conditions with 12∶12 h light dark cycle (lights on at 0700 h) with food and water available *ad libitum*.

### Ethics Statement

All procedures were approved by the UK Home Office under the Animals (Scientific Procedures) Act 1986, under a project licence approved by the local ethics committee.

### Isolated SON

Adult randomly cycling female rats or PND-8 rats were decapitated, their brains were rapidly removed and SONs dissected out in normal Locke's buffer (NL; composition in mM: NaCl 140, KCl 5, 4-(2-hydroxyethyl)piperazine-1-ethanesulfonic acid (HEPES) 10, glucose 10, MgCl 1.2, CaCl_2_ 2.2; pH 7.35, osmolarity between 295 and 300 mOsm). Four SONs were placed in a chamber containing 110 µl buffer, maintained at 35°C. The buffer was replaced every 5 min for 45 min while secretion reached equilibrium, after this, samples were taken every 5 min and stored (−20°C) for subsequent assay of peptide content. At 30 min after starting the experiment, SONs were stimulated with 50 mM K^+^ for 10 min (mM: NaCl 95, KCl 50, HEPES 10, glucose 10, MgCl 1.2, CaCl_2_ 2.2; pH 7.35, osmolarity between 295 and 300 mOsm). After 30 min, the tissue was treated either with 0.2 µM thapsigargin or vehicle (0.1% DMSO) for 10 min. Then, 60 min later, the tissue was treated with a Ca^2+^ channel blocker or vehicle (0.1% DMSO) for 10 min followed by 50 mM K^+^ plus the channel blocker or vehicle for 10 min. The channel blockers used were: L-type, 1 µM nicardipine (Sigma-Aldrich, UK); P/Q-type, 0.2 µM ω-agatoxin IVA (Alomone Labs, Jerusalem, Israel); N-type, 0.5 µM ω-conotoxin GVIA (Alomone Labs, Jerusalem, Israel); R-type, 0.02 µM SNX 482 (Alomone Labs, Jerusalem, Israel).

### Measurement of oxytocin

Oxytocin release was measured by radioimmunoassay as previously described [49] using an antibody (oxytocin 242) kindly provided by Chris Chapman (Babraham Institute, Cambridge, UK). The oxytocin standard was obtained from the National Institute for Biological Standards and Control (Potters Bar, Hertfordshire, UK) and iodinated oxytocin from PerkinElmer LAS Ltd (Beaconsfield, Bucks, UK). The assay had a sensitivity of 0.5 pg, inter-assay variability of 9% and intra-assay variability of 10%. Evoked oxytocin release was calculated by subtracting the total basal release in the five fractions before stimulus from that observed during (two fractions) and directly after (three fractions) the K^+^ stimulus. The effect of channel blockers on the release induced by a second K^+^ stimulus was calculated as the percentage of the response to the first.

### Dissociated SON neuron preparation

Adult female or eGFP-vasopressin rats were decapitated; the SONs rapidly dissected in NL and incubated in oxygenated NL containing 0.5 mg/ml deoxyribonuclease-1 and 1 mg/ml protease X for 20 min at room temperature which was then replaced with NL containing 0.5 mg/ml deoxyribonuclease-1 and 1 mg/ml protease XIV for 20 min. The tissue was then incubated in oxygenated NL for at least 60 min before further manipulation. Single magnocellular neuron suspensions were generated by mechanical tituration. The cell suspension was placed on poly-L-lysine treated coverslips and cells allowed to adhere for 20–30 min before *in vitro* electrophysiology. For immunohistochemistry, cells were fixed *in situ* with 4% paraformaldehyde for 20 min at room temperature.

### Brain sections

Free-floating brain sections were from female adult random-cycling, lactating rats or PND-8 pups which had been deeply anaesthetised (Sagatal, 1 ml *i.p.*) then perfused through the ascending aorta with heparin (5000 U/ml; 300 ml in 0.9% NaCl solution) followed by 300 ml of 4% paraformaldehyde in 0.1 M phosphate buffer (PB, pH 7.4). The brains were removed and immersed overnight in a solution of 0.2% paraformaldehyde and 15% sucrose in 0.1 M PB at 4°C. The tissue was then placed in 30% sucrose in 0.1 M PB and left at 4°C until it had sunk (48 h). The hypothalami were cut coronally with a freezing microtome (40 µm) and rinsed in 0.1 M PB before fluorescence immunocytochemistry.

### Fluorescence Immunocytochemistry

Sections containing the SON from three rats in each group (adult, lactating and PND-8) were distributed so at least one section per rat in each treatment group was exposed to each different antibody combination. In this way, at least 5 SON from 3 different rats were used to analyse each different combination of peptide and one of the five Ca_v_ α_1_-subunit isoforms. Fixed dissociated neurons were washed with 0.1 M PB and processed in situ in multi-well trays.

After washing, sections or dissociated neurons were incubated for 30 min in a blocking buffer consisting of 1% BSA + 0.2% Triton X-100 in 0.1 M PB. They were then incubated with primary antibodies against oxytocin, vasopressin (PS38 and PS41 respectively, a kind gift from Dr Hal Gainer) or the Golgi marker GM130 (1/500, BD Transduction Laboratories, only data for dissociated neurons shown) and one of five Ca_v_ α_1_-subunit isoforms: 1.2, 1.3, 2.1, 2.2 and 2.3 (1/200, 1/100, 1/200, 1/400, 1/200 respectively, Alomone Labs, Jerusalem, Israel) [46], [50]–[52] first for 60 min, then for 48 h at 4°C. After washing in 0.1 PB, sections or dissociated neurons were incubated for 60 min with biotinylated-anti-rabbit IgG (1∶500, Vector Laboratories, UK), followed by 60 min with Alexa 568-streptavidin conjugate and anti-mouse IgG Alexa 488 (1∶1000, Molecular Probes, USA). Both primary and secondary antibodies were diluted in blocking buffer. After further washing, sections or dissociated neurons were mounted using a Mowiol 4–88 (Calbiochem, USA) mounting medium, supplemented with 2.5% DABCO (Sigma). No fluorescent labelling was detected when primary antibodies were omitted or incubated with a five-fold (w/v) excess of control immunogen (provided by the antibody providers) before being exposed to the cells or tissues. All processing was at room temperature.

To analyse cellular localisation of channel subunits in brain slices, fluorescence signals were observed with a Leica upright TCS-NT confocal microscope (Leica Microsystems, Heidelberg, Germany) equipped with an Argon/Krypton laser. Using a x20 Fluotar objective with NA 0.50, each optical layer was scanned four times, which were then averaged. Emissions from each fluorophore were acquired consecutively to ensure no bleed-through or cross-talk. Images were acquired using Leica Confocal Software (Leica Microsystems, Heidelberg, Germany). Optical sections from each SON (n = 5) in each group (adult, lactating and PND-8) were displayed on a computer screen and analysed using ImageJ (http://rsb.info.nih.gov/ij/). The images were converted to 8 bit and the optical density of subunit staining was determined by first making region-of-interest templates using staining for either oxytocin or vasopressin, then applying these to the channel showing the subunit staining and measuring the optical density for the peptide and subunit within them. As different SON sections have different numbers of oxytocin or vasopressin neurons, the optical density of the subunit in regions of interest in different sections was normalising by dividing by the optical density for the peptide in that region and these values were averaged within each treatment group.

To analyse subcellular localisation, fluorescence signals from dissociated neurons were acquired using a Zeiss LSM510 Axiovert confocal laser scanning microscope equipped with argon/krypton lasers. Signals were acquired at 1024×1024 pixels, using a Zeiss Plan NeoFLUAR 1.4 NA x63 oil-immersion objective. Emissions for both fluorophores were again obtained consecutively to avoid channel cross-talk. Images were taken throughout each cell at Nyquist sampling rates and were deconvolved using Huygens software (Scientific Volume Imaging) and the images examined using NIH ImageJ software. To examine co-localisation, residual maps were generated by calculating the residual of each voxel from a linear regression fit of the intensity of each channel within each voxel. The residuals ranged from −1 to 1 (pixels coloured cyan indicate zero residual) with brightness corresponding to the combined intensity of the two channels.

### *In vitro* electrophysiology

SON neurons from adult female or eGFP-vasopressin rats were acutely dissociated and plated on coverslips as described above. The coverslips were used as the base of perfusion chambers (Warner Instruments, Hamden, USA) mounted on an inverted Zeiss Axioskop-2 microscope. Chambers were perfused at 1.5 ml/min with oxygenated medium (mM: NaCl 125; CaCl_2_ 5; HEPES/Na 10; glucose 10; TTX 0.0002; pH 7.4; 300 mOsm; 30°C). Patch pipettes pulled from borosilicate capillary glass had a resistance of 4–5 MΩ when filled with (in mM) CsCl_2_ 125; HEPES 10; EGTA 11; CaCl_2_ 1: ATP-Mg 4; GTP-Tris 0.5; leupeptin 0.1; phosphocreatin 14 and phosophocreatin kinase 0.2 mg/ml; pH 7.2; 290–295 mOsm. Calcium currents were recorded using a patch-clamp amplifier (Axopatch 200 B, Axon Instruments, CA, USA) filtered at 1–2 kHz, digitised at 2–5 kHz (Digidata 1322A; Axon Instruments), and analysed with pClamp v9 software (Axon Instruments).

Currents evoked by a depolarisation to potentials ranging from −60 to +30 mV were normalised against the peak maximum current evoked by a depolarisation to −10 mV. These were used to express the mean normalised conductance (G/G_max_) at each potential (n = 13−15). The data points were fitted using the following Boltzmann equation where V_0.5_ represents half-maximal activation, *k* the exponential slope factor, V the membrane potential, V_rev_ the reversal potential for I_Ca,_ and G_max_ the maximum whole cell conductance.
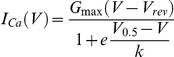



### Statistics

Data were analysed using SigmaStat® software (Systat Software Inc., Richmond, CA, USA) by *t-*tests (paired or unpaired) or, when appropriate, by a one-way ANOVA on ranks followed by Dunn's or Holm-Sidak *post-hoc* tests. Values are expressed as means±S.E.M., and differences were considered significant when *P*<0.05.
